# Incidence of and Risk Factors for Tuberculosis among Cancer Patients in Endemic Area: A Regional Cohort Study

**DOI:** 10.31557/APJCP.2020.21.9.2715

**Published:** 2020-09

**Authors:** Sirinya Nanthanangkul, Supannee Promthet, Krittika Suwanrungruang, Chalongpon Santong, Patravoot Vatanasapt

**Affiliations:** 1 *Doctor of Philosophy Program in Epidemiology and Biostatistics, Faculty of Public Health, Khon Kaen University, Khon Kaen, Thailand. *; 2 *ASEAN Cancer Epidemiology and Prevention Research Group, Khon Kaen, Thailand. *; 3 *Cancer Unit, Srinagarind Hospital, Faculty of Medicine, Khon Kaen University, Khon Kaen, Thailand. *; 4 *Department of Otorhinolaryngology, Faculty of Medicine, Khon Kaen University, Khon Kaen, Thailand. *

**Keywords:** Epidemiology, latent infection, malignancy, screening, tuberculosis

## Abstract

**Background and Objective:**

Cancer is a known risk factor for developing active tuberculosis (TB) disease. The incidence of and risk factors for TB are not known among cancer patients in Thailand. This study aimed to investigate risk factors for TB among cancer patients in an area with endemic TB infections.

**Methods:**

We used the Khon Kaen population–based cancer registry and two TB databases to conduct a retrospective cohort study of cancer patients. From 2001 to 2015, we identified 40,948 eligible cancer patients. Following until 2017, we identified cases of TB diagnosed after cancer diagnosis and analyzed primary cancer site, staging, treatment, and demographic factors. Adjusted incidence rate ratios (adj. IRR) were computed to identify risk factors among a sub–set of cancer types (n = 9,733) using Poisson regression.

**Results:**

Among all cancer patients, 472 cases of TB were diagnosed following cancer diagnosis (cumulative incidence = 1.15%, incidence rate = 421.86 cases per 100,000 patients per year). Among the sub–set of cancer types, 206 cases of TB were found (cumulative incidence = 2.11%, incidence rate = 848.26 cases per 100,000 patients per year). Risk factors for TB among cancer patients were sex (p < 0.001) (male adj. IRR = 1.87, 95% CI: 1.36–2.59), age (p < 0.001) (age >70 adj. IRR = 2.36, 95% CI: 1.56-3.55, compared to age ≤50) and cancer site (p < 0.001). Compared to thyroid cancer, TB infection was more associated with lung cancer without histopathological confirmation (adj. IRR = 6.22, 95% CI: 2.57–15.04). Cancer stage and treatment did not show statistically significant trends.

**Conclusion:**

Old age, male sex, and certain cancer types were independent risk factors for TB in cancer patients. Targeted latent TB screening may be appropriate among high risk groups.

## Introduction

Cancer is a major public health problem worldwide and among the most common causes of morbidity and mortality. It has long been recognized that cancer can cause malnutrition and diminished immune functioning directly from the disease or indirectly via secondary effects. The treatment of cancer, especially chemotherapy, also plays an important role in immunosuppression. This leads to an increased risk of reactivating latent tuberculosis (TB) infections or new TB infection during the clinical course of cancer in endemic areas of the disease. Increased risk of TB infection has been found among certain cancer types, including lung, hematologic, colon, and oral (Cheng et al., 2016; Dobler et al., 2017; Huang et al., 2011; Teng et al., 2019). Thailand is among the 14 countries with the highest burden of TB, with an estimated incidence of 120,000 new cases of TB per year (WHO, 2018; Bureau of Tuberculosis, Department of Disease control Ministry of Public Health, 2017). However, the incidence of and risk factors for TB are not known among cancer patients in Thailand. The purpose of this study was to use a regional population–based cohort of cancer patients to analyze this issue and identify groups at higher risk of TB infection.

## Materials and Methods

*Data Source and Study Design*

We conducted a retrospective cohort study of cancer patients. Cancer patient data was queried from the Khon Kaen population–based cancer registry. This cancer registry includes all cancer diagnoses among the population of Khon Kaen province. TB data was queried from two databases: the Khon Kaen Central Hospital TB Database, which records diagnoses of TB, and the TB database of the Region 7 Office of Disease Prevention and Control, which logs TB cases for the area of Khon Kaen province. The study was approved by the Khon Kaen University Ethics Committee for Human Research (reference number HE 611542).

*Study Cohort*

**Figure 1:**
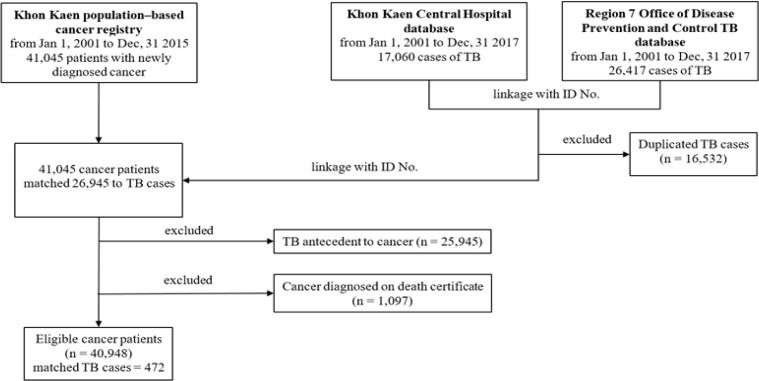
Data Flow Chart

From January 1st, 2001 to December 31st, 2015, new cancer diagnoses were identified with the International Classification of Diseases for Oncology (ICD–O 3rd edition, code C00.0–C80.9) and later converted to ICD–10 for reporting (n = 42,045). The TB databases were then queried for all TB cases (ICD–10 version 2016, code A15.0–A19.9) that were assigned to patients included in the cancer cohort (linked by ID number) between the time period from January 1st, 2001 to December 31st, 2017. After removing duplicates (n = 16,532), a total of 26,945 TB cases were matched to the cancer cohort (Fig. 1). Patients were excluded if the TB diagnosis was made prior to the cancer diagnosis (n = 25,945) or if the cancer diagnosis was not recorded until the death certificate (n = 1,097). 

*Data Extraction*

The primary cancer site, staging, cancer treatment, and demographic data were extracted as independent factors. TB diagnosis and time to diagnosis were extracted as dependent variables. Cancer site was coded as one of seventeen cancer sites, including breast, colorectal, liver and bile duct, lung (with histopathological confirmation), lung (without histopathological confirmation), lymphoma, hematopoietic (leukemia), female organ (cervix, corpus, uterus, ovary, perineum), male organ (penis, prostate, testis), head and neck, skin, thyroid, digestive organ, brain, urinary tract, unknown primary and other cancers. Cancer extent was categorized according to SEER Summary Staging 2000 codes as localized, regional, distant, not applicable, or unknown. Cancer treatment included surgery, chemotherapy, radiotherapy, or combinations thereof. Demographics included sex and age.

*Statistical analysis*

The development of TB was the primary dependent variable for this study. Follow-up of the cohort ended on the date of TB diagnosis, the patient’s death, or December 31, 2017. The cumulative incidence and incidence rate were computed using the Kaplan-Meier method. Chi-squared tests were used to compare TB cases among categorical variables. Risk factors were assessed using Poisson regression analysis to compute adjusted incidence rate ratios also check assumption with the overdispersion testing then it passed this requirement. In the Poisson analysis, the dependent variable was the time to TB diagnosis following cancer or loss to follow–up, with the person–years as the risk offset variable. Factors were included in the initial model if they were found to be p–value < 0.25 from the bivariate analysis or if were found to be essential from reviewed literature. Only certain cancer types were included in the analysis, based on literature review that the cancer type impact to TB (thyroid, lung, lymphoma, hematopoietic, digestive organ, urinary tract cancer), yielding a final analysis sample size of 9,733. STATA statistical software (version 15.0 StataCorp. 2017. College Station, TX: StataCorp LLC) was used in the analysis.

## Results

*Clinical and demographic characteristics of cancer patients*

From January 2001 to December 2015, 40,948 eligible patients were diagnosed with cancer and included in the study. The cohort had a median age of 60 years and had slightly more males (51.3%) (Table 1). Following until 2017, 472 post–cancer diagnoses of TB were identified (cumulative incidence 1.15%). Post–cancer TB diagnosis was more common in males (cumulative incidence 1.41%) and nearly equal among age groups (p = 0.990). Cumulative incidence of post–cancer TB differed among type of cancer, with the highest proportions found in patients with lymphoma (cumulative incidence 3.05%), hematopoietic (2.93%) and lung cancer (without histopathological confirmation) (2.15%) (Table 2). Considering the staging, cumulative incidence was highest among localized tumors (1.65%), followed by regional (1.15%), and distant metastasis (0.93%). Among treatments, chemotherapy had the highest cumulative incidence of TB (1.84%). 

*Incidence rate for TB among cancer patients*

Among all cancer patients, 472 post–cancer TB cases were identified from a total of 111,886 person–years of follow–up (incidence rate 421.86 cases per 100,000 patients per year). Considering a sub–set of cancers shown in literature to be associated with higher risk of active TB infection (Table 3), 206 cases of post–cancer TB were found (cumulative incidence = 2.11%) among 24,285 person–years of follow–up (incidence rate = 848.26 cases per 100,000 patients per year).

*Risk factors for TB among cancer patients*

There was a statistically significant difference of incidence rate ratios (IRRs) among age group, gender and cancer type. All independent variables were included into the risk factor analysis. However, only a sub–set of cancer types were included (Table 3) (n = 9,733). The highest adjusted IRRs (adj. IRR) were for age > 70 year (adj. IRR : 2.36, 95% CI: 1.56–3.55), and males (adj. IRR : 1.87, 95% CI; 1.36–2.59). For cancer types, we found lung cancer (without histopathological confirmation), lymphoma, hematopoietic, digestive organ cancer, urinary tract cancer and lung cancer (with histopathological confirmation) to have higher adj. IRR at statistically significant levels. Incidence of post–cancer TB was not statistically significantly different among cancer staging, while the trend for types of cancer treatment was unclear (Table 3).

**Table 1:** Demographic Characteristics and Post–Cancer TB Diagnosis Status of Cancer Patients

		Cancer patients with no TB		Post–Cancer TB diagnosis		
Characteristics	Cancer Patients (n)	n	%	n	%	*P*–value
Age						0.99
<= 50	10,789	10,664	98.86	125	1.16	
51–60	9,972	9,859	98.87	113	1.13	
61–70	10,695	10,569	98.82	126	1.18	
> 70	9,492	9,384	98.86	108	1.14	
Total	40,948	40,476	98.85	472	1.15	
Median	60	60		60 (17,89)		
(Min, Max)	(10, 89)	(10, 87)		58.94 (14.70)		
Mean	58.92	58.92				
(SD)	(15.15)	(15.15)				
Sex						<0.001
Male	21,014	20,718	98.59	296	1.41	
Female	19,934	19,758	99.12	176	0.88	
Total	40,948	40,476	98.85	472	1.15	

**Table 2:** Clinical Characteristics and Post–Cancer TB Diagnosis Status of Cancer Patients

		Cancer patients with no TB		Post–Cancer TB diagnosis		
Characteristics	Cancer Patients (n)	n	%	n	%	p–value
Cancer type						< 0.001
Liver and Bile Duct CA	12,948	12,888	99.54	60	0.46	
Female organ CA	3,701	3,671	99.19	30	0.81	
Breast CA	3,647	3,619	99.23	28	0.77	
Colorectal CA	3,224	3,190	98.95	34	1.0.5	
Lung CA (no histology)	2,656	2,599	97.85	57	2.15	
Head and neck CA	2,376	2,340	98.48	36	1.52	
Hematopoietic	1,844	1,790	97.07	54	2.93	
Unknown primary CA	1,591	1,566	98.43	25	1.57	
Skin CA	1,308	1,285	98.24	23	1.76	
Lung CA (histology)	1,253	1,231	98.24	22	1.76	
Digestive organ CA	1,207	1,194	98.92	13	1.08	
Lymphoma	1,114	1,080	96.95	34	3.05	
Thyroid CA	963	954	99.07	9	0.93	
Urinary tract CA	902	885	98.12	17	1.88	
Male organ CA	852	836	98.12	16	1.88	
Other CA	831	822	98.92	9	1.08	
Brain CA	531	526	99.06	5	0.94	
Total	40,948	40,476	98.85	472	1.15	
Cancer stage (by extent)						
Localized	2,483	2,442	98.35	41	1.65	
Regional	5,834	5,767	98.85	67	1.15	
Distant	8,066	7,991	99.07	75	0.93	
Not applicable	2,381	2,309	96.98	72	3.02	
Unknown stage	22,184	21,967	99.02	217	0.98	
Total	40,948	40,476	98.85	472	1.15	
Cancer treatment						0.018
No treatment	22,361	22,121	98.93	240	1.07	
Only SURG	6,685	6,600	98.73	85	1.27	
Only CMT	2,942	2,888	98.16	54	1.84	
Only RT	1,841	1,822	98.97	19	1.03	
SURG+CMT	4,148	4,110	99.08	38	0.92	
SURG+RT	1,304	1,290	98.93	14	1.07	
CMT + RT	824	813	98.67	11	1.33	
SURG+CMT+RT	843	832	98.7	11	1.3	
Total	40,948	40,476	98.85	472	1.15	

**Table 3:** Incidence Rate Ratios (IRRs) for TB by Risk Factor among Cancer Patients Based on Multiple Poisson Regression (n = 9,733)

Variables	Cancer Patients (n)	TB cases (n)	Person time (person–year)	IR (100,000 patients per year)	Crude IRR	Adjusted IRR	95%CI adjusted IRR	p–value
Age								<0.001
<= 50	2,711	52	11,470	453.31	1	1		
51–60	2,177	52	5,220	996.94	2.2	1.63	1.09–2.43	
61–70	2,571	51	4,760	1070.52	2.36	1.52	1.01–2.27	
> 70	2,334	51	2,830	1799.79	3.97	2.36	1.56–3.55	
Total	9,733	206	24,280					
Gender								<0.001
Female	5,796	150	13,110	426.91	1	1		
Male	3,937	56	11,170	1343.19	3.15	1.87	1.36–2.59	
Total	9,733	206	24,280					
Cancer type*								<0.001
Thyroid CA	954	9	6,900	130.53	1	1		
Lung CA**	1,231	22	1,460	1506.11	11.54	5.48	2.16–13.91	
Lymphoma	1,080	34	3,770	901.36	6.91	4.15	1.62–10.61	
Hematopoietic	1,790	54	5,250	1027.95	7.88	3.76	1.46–9.73	
Digestive organ CA	1,194	13	1,430	908.34	6.96	3.9	1.51–10.12	
Urinary tract CA	885	17	3,170	535.85	4.11	2.27	0.91–5.67	
Lung CA***	2,599	57	2,300	2478.22	18.99	6.22	2.57–15.04	
Total	9,733	206	24,280					
Cancer stage (by extent)								0.476
Local	225	2	1,390	143.78	1	1		
Regional	843	14	1,910	733.02	5.1	2.59	0.58–12.54	
Distant	2,196	29	2,310	1257.79	8.75	2.69	0.63–11.54	
Not applicable	2,033	67	6,340	1056.48	7.35	2.83	0.65–12.32	
Unknown	4,436	94	12,340	761.96	5.3	2.24	0.54–9.28	
Total	9,733	206	24,280					
Cancer Treatment								
Only SURG	1,253	19	5,180	367.08	1	1		0.05
No treatment	5,184	120	7,550	1587.26	4.32	1.63	0.84–3.05	
Only CMT	1,782	44	5,470	804.51	2.19	0.94	0.47–1.88	
Only RT	459	7	960	730.09	1.99	1.12	0.43–2.89	
SURG+CMT	346	4	1,220	328.76	0.9	0.47	0.16–1.40	
SURG+RT	401	4	2,890	138.44	0.38	0.91	0.28–2.97	
CMT + RT	258	7	850	822.05	2.24	0.97	0.37–2.55	
SURG+CMT+RT	50	1	160	613.13	1.67	1	0.13–7.63	
Total	9,733	206	24,280					

Remark; IRR, Incidence rate ratio; 95% CI , 95% confidence interval; 1, Reference group; SURG, surgery; CMT, chemotherapy; RT, Radiotherapy; *, Included only 7 cancer types in the multiple poisson regression; **, Lung cancer with histological confirmation; ***, Lung cancer without histological confirmation

## Discussion

To our knowledge, this study is the first to assess association of tuberculosis with risk factors among cancer patients in Thai population. In our study, we found an incidence rate of 421 to 848 per 100,000 per year, depending on the group of cancer types. The main findings showed that the major factors associated with higher incidence of TB included aging, male gender, lung cancer, hematopoietic, lymphoma, and digestive organ cancers. Advanced age changes the immune system and, with time, leads to increased vulnerability to infectious diseases (Nikolich-Žugich, 2018). Immunosenescence affects various cell types in the bone marrow and the thymus, mature lymphocytes in the peripheral blood and secondary lymphatic organs, and also elements of the innate immune system (Weiskopf et al., 2009). Therefore, elderly cancer patients, which were prone to immunosuppression, experienced higher incidences of TB compared to the young age group. Considering gender, male is indeed a risk factors for TB. Biological mechanisms may actually account for a significant part of this difference between male and female susceptibility to TB (Lienhardt et al., 2005; Neyrolles and Quintana-Murci, 2009). Females naturally present more effective immune responses and pathogen resistance, such as vaccination and infection, than males. Gender differences in other risk factors of TB may indirectly impact the male–to–female ratio of TB, such as smoking, alcohol consumption and concurrent HIV infection. Males could have differing levels of M. tuberculosis exposure compared to females because of differences in social roles and activities. Males tend to travel, have direct contact, and spend time in confined places that increase transmission of infectious diseases (Nhamoyebonde and Leslie, 2014) 

Cancer patients are increased risk of developing TB. In particular, systemic infections can be a risk when the host immune responses fail to uncontrol bacterial replication and active disease ensues (Delogu et al., 2013). Healthy individuals can harbor latent TB infections for their entire lives, but in around 5 to 15 percent of infected individuals, TB disease can be reactivated (Vynnycky and Fine, 1997; Kiazyk and Ball, 2017). Immunocompromised individuals are at increased risk of reactivation of latent TB infections, which includes patients hematological malignancies and patients undergoing immunosuppressant cancer therapies such as chemotherapy (Dobler et al., 2017). Our study found that certain cancer types were associated with higher risk of TB, including lung cancer, lymphoma, digestive organ cancer, hematopoietic and urinary tract cancer. For lung cancer, it is possible for misdiagnosis due to mimic finding on the chest. The first case of coexistent TB and lung cancer was reported by Bayle in 1810. (Bayle, 1810; Singh et al., 2009; Christopoulos et al., 2014) It has been well documented that lung inflammation and fibrosis from TB could induce genetic damage, which may increase the risk of lung cancer. (Liang et al., 2009) Reverse causality is also possible, as occult lung cancer may cause TB infection and provoke reactivation of latent TB by weakening the local immunity. (Society, 2000; Wu et al., 2011). 

For lymphoma, it is likely that TB infection affected bone marrow suppression. Our results were consistent with several studies in which lymphoma increased the risk of the development of active TB. (Cheng et al., 2017; Ganzel et al., 2019) The incidence of TB in Non–Hodgkin lymphoma (NHL) patients is 35 times higher than in the general population. Furthermore, interleukin 10 (IL–10), which is increased in patients with NHL, has been implicated in the reactivation of TB. The serum concentration of IL–10 has been found to be higher in patients with NHL, which may lead to decreased cell mediated immunity resulting in activation of latent TB (Hashmi et al., 2017) Hematopoietic cancer was related to TB disease developing. In this study, the cancer type with the highest proportion of patients receiving no treatment was hematopoietic cancer (59.1%; data not shown). Therefore, TB infection may affect the disease itself. Other studies have shown the relative risk of TB disease in hematologic malignancy patients is 2–40 times as compared with the general population (Anibarro and Pena, 2014). Not only the disease itself but also the robust chemotherapy or aggressive therapeutic procedures used to suppress the immune system in hematological cancer patients may lead to TB in these patients and the hematological cancer patients bone marrow or stem cell recipients also tended to have TB as well (Wu et al., 2011). In addition, digestive organ cancer has been shown to be a risk factors for the development of TB in our study. It was consistent with Kim et al., which found that immune suppression by the tumor increased vulnerability to reactivation of TB (Kim et al., 2008). Several studies from Taiwan and Korea have also identified gastric cancer as a significant risk factor for TB (Fang et al., 2015; Jung et al., 2016). The digestive organ in this study included esophagus, stomach, small intestine and other ill–defined digestive organs. As these organs are important to nutrition, digestive organ dysfunction can lead to malnutrition and opportunistic infection. Interestingly, higher incidence of TB was associated with urinary tract cancer in this study. The incidence rate ratios among urinary tract cancer patients in this study (2.27) were similar to those in bladder cancer patients in Taiwan (1.38) (Wu et al., 2011). 

In our study, we used surgery as a reference group in analysis of the effects of treatment, as it was least likely to affect the immune system when comparing to other modalities and to avoid selection bias of using no treatment. Although chemotherapy is an immunosuppressant, we found no significant difference for risk of TB according to cancer treatment. This result may have been due to a limitation of this study, in which the curative and palliative roles of treatment could not be differentiated.

These findings suggest increased TB screening may be necessary in high risk cancer patients (lung cancer, lymphoma, hematopoietic and digestive organ cancer) and older age group (> 70 year) . According to published literature, other types of cancer, such as head and neck cancer and breast cancer, should also be considered for increased TB screening (Cheng et al., 2017). This study adopted the retrospective cohort study for the burden of cancer with TB demonstration. Population–based of cancer registry and TB in the same define population was analyzed for achieved goal. Furthermore, the study period that is long enough to illustrate the change in its trends.

This study reports the incidence rate of TB among cancer patients in a TB endemic area. Risk factors associated with TB among cancer patients included older age group, males, and certain types of cancer, especially lung cancer, lymphoma, hematopoietic cancer, and digestive organ cancer. Increased caution and awareness may be necessary among cancer patients to reduce spread and morbidity. We recommend TB surveillance and prevention plans with emphasis on cancer patients. Increased screening for latent TB infections may also be necessary during cancer treatment.
